# AI-Driven Combination Therapy for Counteracting Dysregulated Genes in Lung Adenocarcinoma: Contribution-Aware Metaheuristic for Drug Repurposing

**DOI:** 10.3390/ph19050748

**Published:** 2026-05-09

**Authors:** Sajjad Nematzadeh, Arzu Karaul

**Affiliations:** Software Engineering Department, Engineering and Natural Sciences Faculty, Istanbul Topkapi University, Istanbul 34087, Türkiye; arzukaraul@gmail.com

**Keywords:** lung adenocarcinoma, drug repurposing, multi-drug combinations, meta-heuristic search, drug–gene interaction networks

## Abstract

**Background/Objectives**: Lung adenocarcinoma (LUAD) is molecularly heterogeneous and often requires rational drug combinations rather than single-agent therapy. Many computational repurposing methods use global signature matching or network scores, but they often treat dysregulated genes equally and optimize a single scalar objective. This study aimed to develop a contribution-aware computational framework for prioritizing repurposed multi-drug combinations that counteract LUAD driver modules; **Methods**: Ten LUAD driver scenarios were curated from the LUAD and non-small cell lung cancer literature and encoded as gene-level counteraction vectors across 44 unique genes. Direction-aware drug–gene interactions from the Comparative Toxicogenomics Database were processed into a weighted contribution matrix. A genetic algorithm was then used to search for small combinations of up to six drugs. The fitness function combined mean absolute error with terms for waste, mismatch, entropy, coverage, combination size, and optional cost. Orthogonal computational support was assessed using CLUE/Connectivity Map transcriptomic reversal analysis; **Results**: After filtering and optimization, 42 drugs and chemicals remained as candidate components across the scenarios. Increasing the combination size from one to three drugs usually reduced the mean absolute error, whereas larger combinations provided more limited gains. Compared with an MAE-only baseline, the full contribution-aware objective improved or preserved MAE in 54 of 60 scenario–drug-count comparisons. Drug and gene clustering identified interchangeable candidate groups and shared mechanisms across LUAD scenarios. CLUE-based analysis provided strong or moderate transcriptomic reversal support for several prioritized compounds; **Conclusions**: The proposed framework provides a transparent, scenario-based method for prioritizing repurposed drug combinations in LUAD. The results are computational and hypothesis-generating. They should guide future experimental testing, not clinical treatment decisions.

## 1. Introduction

LUAD is the most common form of NSCLC and a major cause of cancer death worldwide. In 2024, over 234 K new cases and 125 K deaths of LUAD have been reported [1]. Therapy has advanced with targeted inhibitors for key drivers (*EGFR*, *ALK*, *ROS1*, *BRAF*, *RET*, *NTRK*, *MET*, HER2, *KRAS G12C*) and with immune checkpoint blockade. Examples include first-line osimertinib for EGFR-mutant disease and pembrolizumab for PD-L1-high tumors, which improve survival over prior standards [2,3]. New agents such as the *KRAS* G12C inhibitor sotorasib expand options for molecular subsets [4]. However, intratumor heterogeneity and acquired resistance limit durability and underscore the need for rational combination strategies and robust repurposing frameworks [5].

Drug repurposing can deliver new treatment options faster and at lower risk by starting with agents with established safety, pharmacology, and manufacturing pathways. It allows researchers to match existing drugs to disease biology using scalable in silico tools such as signature matching and network reasoning, then confirm the best hypotheses in focused experiments [6]. In oncology, repurposing supports precision strategies for molecular subgroups and for rational combinations that address resistance and pathway cross-talk [7]. Recent reviews highlight that systematic repurposing complements de novo discovery, improves translational efficiency, and can shorten the path to clinical testing when evidence is strong [8].

In this study, we propose a contribution-aware framework to repurpose drugs and chemicals for LUAD. First, we briefly review recent works in drug repurposing, with emphasis on NSCLC and LUAD. Next, we present a semi-personalized view using 10 literature-based scenarios that capture dysregulated genes and expression patterns known to drive LUAD. These scenarios reflect common molecular conditions in the disease. In practice, this module can be replaced with patient-specific causality and dysregulation profiles to guide individualized therapy design. Unlike our previous Biomolecules 2025 review [9], this manuscript does not aim to re-review LUAD biology. It focuses on the new computational framework, CTD processing, contribution-aware fitness design, GA implementation, MAE-only comparison, CLUE-based orthogonal support, and the resulting drug-combination hypotheses.

### 1.1. Drug Repurposing Landscape

Repurposing strategies can be categorized into two broad scopes: target-centric and disease-centric. Target-centric approaches begin with a known molecular target and ask whether its existing modulators can treat another disease that shares the same mechanism. These approaches are precise but depend on clear target-disease causality [10]. Disease-centric approaches begin with the disease state and search for drugs that reverse or modulate its global signatures (for example, transcriptomic patterns). Although this approach is broader, it can be sensitive to data quality and context [6].

At the therapy level, drug treatment can be classified into two categories: single drugs and rational combinations. At the therapy level, single-drug repurposing provides simpler safety and dosing. At the same time, rational combinations aim to cover complementary pathways, boost efficacy, and curb resistance, but require principled synergy models and careful toxicity management [11].

Core data for repurposing integrates disease biology with evidence on drugs. Disease biology comes from multi-omics and phenotypes, including transcriptomes and other profiles from public resources such as GEO and LINCS. These resources capture the molecular state of the disease and its perturbation space [12,13]. Drug evidence includes measured or inferred effects (e.g., perturbational signatures), known targets and mechanisms (from curated chemogenomic knowledge bases), and safety attributes such as adverse events and interactions [14,15]. Combining these layers (omics/phenotypes to define what should be countered, and targets/effects/safety to define what drugs can do) supports systematic, testable hypotheses for single agents and combinations in diseases such as LUAD.

In silico repurposing methods are divided into three main families: signature matching, network/pathway reasoning, and ML/AI models. Signature matching compares a disease’s differential expression to drug-induced profiles and prioritizes agents that reverse the signal. It is scalable and hypothesis-driven but sensitive to platform and context effects [6,13]. Network and pathway reasoning propagates evidence over protein, gene–drug, and pathway graphs to capture context and polygenic mechanisms. The performance depends on network completeness and edge quality [16,17,18]. Machine-learning and AI models integrate heterogeneous features (omics, networks, chemistry, indications) to predict drug–disease links and potential synergies. While they offer high coverage, they raise challenges in interpretability and dataset shift [19,20].

In silico repurposing is a broad, fast approach: it screens many candidates, integrates diverse data, and generates testable hypotheses at low cost [6]. Its limitations include data noise, model bias, and context dependence, which can reduce precision when transitioning to experiments [19]. In vitro studies are both specific and translational, as dose–response and synergy assays quantify real cellular effects, which help to shape dosing and combinations [21,22]. They are slower and costlier, and results may not translate to in vivo pharmacology without careful follow-up on the mechanism, pharmacokinetics (PK)/pharmacodynamics (PD), and safety.

Rational combination design seeks to cover complementary pathways and resistance routes while minimizing overlapping mechanisms and toxicities. In practice, this means pairing agents whose targets lie in distinct (but disease-relevant) modules so the drugs jointly counteract the tumor network. At the same time, it tends to avoid redundant actions that amplify adverse effects or narrow the dosing window [17,23]. Modern screening and analysis frameworks (e.g., matrix designs and standardized synergy scoring) facilitate the ranking of such pairs. However, most computational methods still optimize reversal or synergy scores that implicitly treat all disease genes as equally important, ignoring heterogeneous gene contributions and context [21,24]. This gap motivates us to propose contribution-aware strategies that weight targets by their impact and balance efficacy with safety at the combination level.

An evaluation ladder links computation to translation. First, in silico scoring prioritizes candidates by either reversing disease signatures or matching targets [6]. Next, cell models test single-agent and combination activity with standardized dose–response and synergy analyses to confirm effect size and robustness [21]. Mechanistic assays then verify pathway engagement and identify biomarkers that predict response and inform dosing strategies. Finally, preclinical studies extend to pharmacology and safety and emerging clinical signals (e.g., early response and tolerability) [25].

Most repurposing pipelines optimize reversal or network scores that treat dysregulated genes as if they contribute equally, thereby missing the unequal impact of clustered driver and effector genes in disease. We address this gap by weighting genes based on their contribution to the disease signature and clustering candidate drugs into functional sets based on targets and perturbational effects. We then search for combinations that counteract high-contribution gene clusters as much as possible while constraining redundancy and expected toxicity. This contribution-aware design builds on signature matching and network reasoning but shifts the objective from gene-agnostic reversal to weighted, mechanism-informed control of LUAD modules [13,17,21,26].

Compared with signature-matching methods, our framework not only searches for global transcriptomic reversal but optimizes gene-level counteraction within defined LUAD scenarios. Compared with network-based methods, it uses direction-aware drug-gene effects rather than network proximity alone. Compared with scalar synergy models, it evaluates each drug’s contribution to each gene and penalizes waste, mismatch, and unnecessary combination size.

### 1.2. LUAD and Scenarios for Counteraction

To connect LUAD biology to the computational framework, this study defines ten literature-based scenarios and encodes each one as a gene-level counteraction module. These scenarios are not intended to provide a full clinical review of LUAD. Instead, they define the biological search space used by the optimizer. Each scenario includes a small set of genes and a desired direction of change, where “−” denotes suppression of an activated oncogenic signal and “+” denotes restoration of a reduced protective function. A compact summary is provided in Table 1, while the full narrative description of the scenarios is moved to Appendix A.

These ten scenarios provide interpretable driver-centered modules for the optimization framework. They do not capture the full heterogeneity of LUAD, but they enable the search process to operate with explicit gene-level directional goals. In practice, the same framework could use patient-specific molecular profiles instead of literature-defined scenarios.

**Figure 1:**
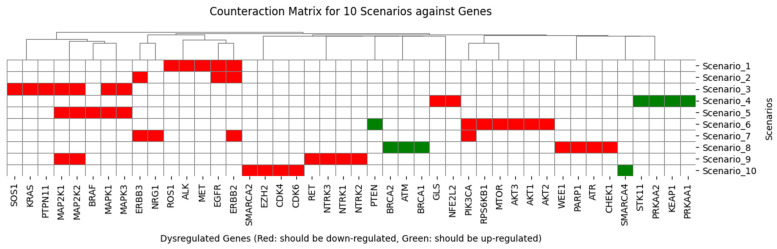
Counteraction matrix of genes associated with LUAD involved in scenarios. Red cells aim to downregulation and green cells to upregulation.

Figure 1 illustrates genes associated with LUAD, organized by scenario. Each scenario lists dysregulated genes. Red cells mark genes we aim to inhibit, and green cells mark genes we aim to upregulate. This matrix feeds the combinatory search over drugs and non-drug substances to generate testable therapeutic clues in this study.

### 1.3. Scenario Reproduction Policies

For each scenario, we selected genes by manual curation from recent reviews of LUAD and NSCLC and from key primary studies on that driver. We included both actionable targets (such as mutant kinases) and well-supported downstream effectors. Then, we kept membership as a simple binary choice (a gene is either in the scenario or not, without extra weights). For each included gene, we defined a desired direction of change: −1 for genes that should be downregulated (e.g., oncogenes or activated effectors) and +1 for genes that should be upregulated (e.g., tumor suppressors or depleted pathway components). All counteraction magnitudes were set to ±1. Here, we did not use larger values. When a gene could plausibly belong to more than one scenario, we kept the same desired direction across scenarios and allowed overlap only when the literature supported a consistent role.

In short, most existing and classical repurposing methods match global expression signatures without weighting genes. Also, network diffusion/pathway approaches do not weight gene contributions. Moreover, synergy screens use a single scalar score that optimizes only a scalar synergy score. In contrast, the proposed method of this study defines scenario-based counteraction vectors that represent semi-personalized driver modules and can be replaced in practice by patient-specific profiles. We then optimize combinations using a contribution-aware fitness that works at the per-drug, per-gene level and balances the desired counteraction with waste, mismatch, entropy, coverage, combination size, and cost. This design links high-level clinical scenarios to low-level gene regulation, making the search more interpretable and controllable than in classical approaches.

Because LUAD is molecularly heterogeneous and many candidate compounds can emerge from computational screening, additional prioritization layers are needed before biological follow-up is considered. Approaches that combine gene-level directionality, scenario-based evaluation, and transparent ranking rules can help reduce noise and improve the interpretability of candidate selection. At the same time, primary computational predictions should be examined with an independent secondary layer. For this reason, orthogonal computational validation was used in this study to assess whether prioritized drugs also show external transcriptomic reversal support. This added layer does not replace experimental or clinical validation, but it can provide stronger support for compounds that remain consistent across independent evidence sources.

## 2. Results and Discussion

The GA is an iteration-constrained metaheuristic that searches for near-optimal solutions within a fixed number of epochs. Because of randomized initialization, parent selection, crossover, and mutation, outcomes can be different across runs. Repeated runs may also yield duplicate combinations. Across 10 runs and 10 scenarios, the search produced multiple raw solutions with drug counts between 1 and 6. Duplicate combinations were removed within each scenario.

**Figure 2:**
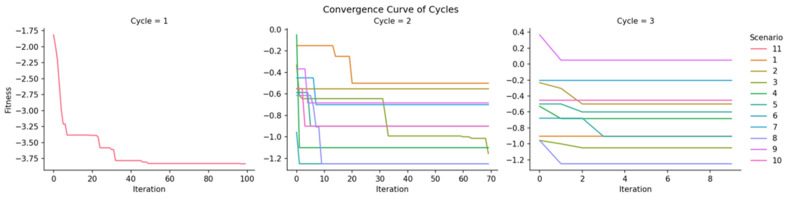
Convergence curves of the meta-heuristic in three cycles.

Figure 2 shows the convergence curves for the three cycles. The first cycle removes drugs that are ineffective or have adverse effects. Because the first curve plateaus around iteration 50, the maximum iterations for Cycle 2 were set to 70. In some Cycle 3 scenarios (particularly Scenarios 7 and 9), reduced fitness gains were observed following a literature review of Cycle 2 survivors. Some chemicals may provide better local counteraction for a small subset of genes, even though they are reported to be toxic, oncogenic, and harmful overall.

**Figure 3:**
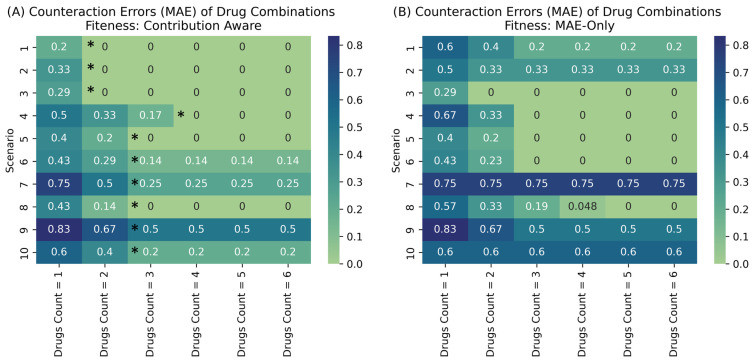
MAE of best-fit drug combinations across combination sizes for dysregulated genes in each scenario (**A**) for the proposed method and (**B**) for MAE only fitness. Asterisks (*) mark the Pareto frontier for each scenario in (**A**).

We filtered the raw solutions based on the lowest MAE and the fewest number of drugs. In Figure 3, an MAE of 0 implies that the drugs fully match the counteraction vector. Values above 0 indicate partial gene coverage. Multiple runs at a fixed combination size can yield multiple solutions. Next, we clustered genes, drugs, and regulatory directions to remove redundancy.

Across the ten LUAD scenarios, Figure 3A showed a similar pattern. When we increased the regimen size from one to three drugs, the MAE usually dropped clearly. However, expanding the regimen size did not help to gain more. Scenarios with broad counteraction vectors and many targets need larger combinations while still keeping a higher residual MAE. More focused scenarios with fewer genes can often be controlled with only two or three drugs. We also found that upregulation targets are harder to satisfy than inhibition targets, which leads to more mismatches in scenarios dominated by depleted tumor suppressors or immune pathways. The coarse global scenario mainly favors broadly acting drugs and has weaker MAE, so it serves as a filter rather than a final solution. Overall, these results suggest that mechanism-focused combinations are sufficient for many LUAD modules. On the other hand, densely dysregulated modules benefit from richer regimens and contribution-aware optimization.

To assess whether the contribution-aware composite objective improved optimization beyond the MAE term alone, we compared the full-fitness search with an MAE-only baseline across all 10 scenarios, allowed combination sizes from 1 to 6, and the same conditions and parameters. Across the 60 scenario drug-count comparisons shown in Figure 3 (10 scenarios × 6 drug-count settings), the full-fitness objective produced lower MAE in 27 comparisons and the same MAE in 27 comparisons. Thus, the contribution-aware objective improved or preserved MAE in 54 out of 60 comparisons (90%). Only 6 comparisons showed higher MAE, mainly in Scenario 6 and one drug-count setting in Scenario 4, suggesting that the added waste, mismatch, coverage, and parsimony terms usually improved or maintained the primary fit while occasionally introducing a controlled trade-off.

**Figure 4:**
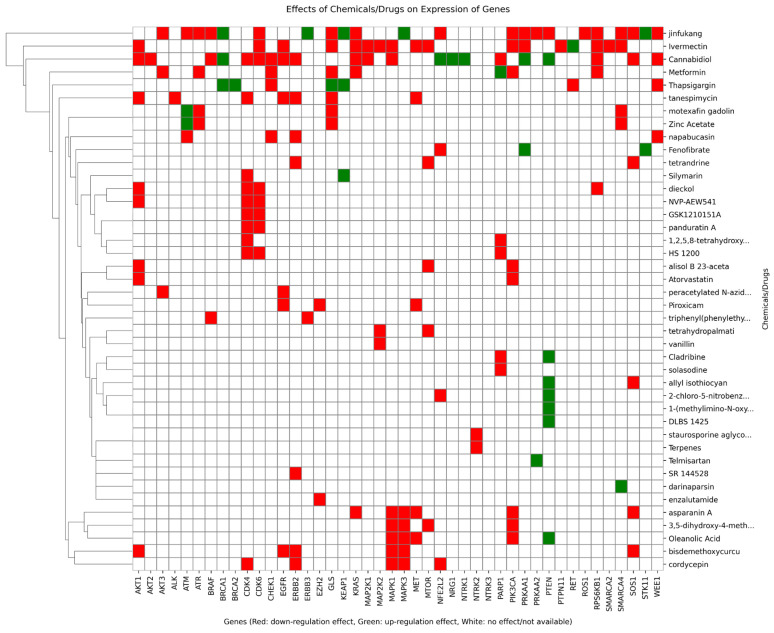
Expression effects of drugs on genes. Red cells aim to downregulation and green cells to upregulation.

Figure 4 illustrates the effects of all chosen drugs on whole genes in all scenarios. The plot is hierarchically clustered and shows potential similarities based on regulation effects. Taking all scenarios into account, Table 2 reports the unique gene clusters and the drug clusters that counteract them.

### 2.1. Hypothetical Drug Combinations

In addition to the cost term included in the fitness function, Table 2 lists alternative compounds for each scenario. Drug groups are shown in the Group column. Each group contains one or more compounds that may provide similar directional support for the corresponding gene cluster under the current computational framework. Thus, the grouped entries should be interpreted as complementary hypothesis-generating options rather than fixed or clinically actionable regimens. For example, in Scenario 4, fenofibrate represents Group A support for *NFE2L2* inhibition and *PRKAA1*/*STK11* upregulation, whereas Group B contains several alternatives reported to inhibit *GLS*. Groups C and D similarly represent complementary options for *KEAP1* and *PRKAA2* support.

### 2.2. Hypothesis-Generating Reference

The prioritized compounds include agents with different levels of translational maturity, including approved drugs, investigational oncology candidates, tool compounds, and natural or mixture-based products. These results should therefore be interpreted as hypothesis-generating rather than treatment recommendations. To reduce the length and keep the main text focused on the proposed framework, detailed compound-level literature context and orthogonal validation notes are provided in Appendix B.

Table 3 summarizes the orthogonal CLUE-based transcriptomic reversal support categories assigned to the prioritized compounds. These categories were derived using the current mapping and filtering framework and should be interpreted with caution. In this context, the “not supported” group indicates that no usable CLUE-based support was assigned under the current analysis, but it does not imply a lack of biological activity, because some compounds may be absent from the CLUE resource, may not have a direct perturbagen match, or may be represented only indirectly through related compounds. In addition, some supported entries were identified through mapped CLUE representatives rather than exact compound-level matches. Because this study focused on combination therapy rather than single-agent selection alone, the complementary role of each compound should also be considered, as a compound with limited direct orthogonal support may still contribute meaningfully within a broader multi-compound therapeutic strategy.

At the pathway level, several recurring patterns were observed. Tanespimycin was detected primarily in RTK-related modules, consistent with HSP90-dependent stabilization of oncogenic signaling proteins. Ivermectin and bisdemethoxycurcumin appeared in MAPK- and PI3K-related modules, suggesting broad pathway-level counteraction. DDR-related scenarios selected compounds linked to *PARP1*, *ATR*, *CHEK1*, and *WEE1* control. In the *SMARCA4*-deficient scenario, the selected candidates mainly reflected epigenetic and cell-cycle vulnerabilities.

## 3. Materials and Methods

### 3.1. Drug–Gene Interaction Data

This study utilized the CTD, a database of drug–gene interactions [27]. The dataset was subset to genes involved in the LUAD scenarios defined in this work. CTD annotates interactions with direction-of-effect labels. We treated the following labels as upregulation:

- increases^expression
- increases^stability
- increases^abundance
- decreases^degradation
- decreases^ubiquitination

We treated the following labels as downregulation:

- decreases^expression
- decreases^stability
- increases^degradation
- decreases^abundance
- increases^ubiquitination

Because CTD aggregates evidence from multiple sources, some drug–gene pairs appear with conflicting directions (for example, the same drug reported to both increase and decrease a given gene). As a preprocessing step, we applied a regulation-bias procedure that estimates the direction probability from the frequency of reported directions across studies. The resulting bias score was then used to resolve conflicts and to weight each drug–gene effect in downstream analyses. Ambiguous pairs with insufficient directional evidence were handled conservatively according to this weighting scheme. Accordingly, CTD-derived drug–gene effects in this study should be interpreted as aggregated evidence signals rather than LUAD-specific quantitative response measurements.

### 3.2. Toxicity/Carcinogenic Cost Management

Not all chemicals and drugs in CTD are safe. Because the list is large, we used a cyclic filter to remove harmful or irrelevant items. In Cycle 1, we ran a unified search (Scenario 11) to drop irrelevant materials. We then applied penalty-based screening from four sources:

- The International Agency for Research on Cancer (IARC) Monographs for carcinogenicity [28] (Supplementary File S1).
- The NTP Report on Carcinogens and the European Chemical Agency (ECHA) harmonized classification and labelling [29] (Supplementary File S2).
- The PAN International consolidated list of banned pesticides [30] (Supplementary File S3).
- We also annotated withdrawn status and clinical phase from ChEMBL [31] (Supplementary File S4).

The surviving set advanced to Cycle 2 (Supplementary File S5), where we performed focused literature curation. Cycle 3 yielded the final candidates reported in the Results and Discussion section. This filtering framework is modular and can be extended with patient-specific constraints.

### 3.3. Connectivity Map for External Validation

Orthogonal computational validation was performed with publicly available perturbational transcriptomic data from the Connectivity Map (CMap) resource through the CLUE platform. This resource was developed by the Broad Institute within the NIH LINCS program and provides large-scale gene expression profiles of chemical and genetic perturbations across multiple cellular contexts. The L1000 assay measures 978 landmark transcripts and uses these measurements as the basis for transcriptomic signature analysis in the CMap framework [13]. In this study, CLUE was used only as an external validation layer for prioritized LUAD drug candidates. A signed LUAD disease query, comprising upregulated and downregulated gene sets, was compared with reference perturbational signatures in the CMap collection. Connectivity scores reported by the platform were then interpreted in the context of transcriptomic reversal: negative connectivity indicated support for reversing the disease-associated signature, whereas positive connectivity indicated similarity to that signature. This analysis was used to provide orthogonal computational support and was not treated as experimental, clinical, or synergy validation.

### 3.4. Methodology

In the proposed method of this study, we evaluate candidate drug sets by summing single-drug gene-direction effects, clipping them to form an overall drug–effect vector. Primary fit is the MAE to the desired counteraction vector. We then make the score contribution-aware using an attribution matrix that allocates each drug’s share of the net per-gene effect. Secondary terms shape the objective: a waste penalty for unused or canceled effects, a mismatch penalty for opposite-sign influence, an entropy regularizer to avoid over- or under-concentration of contributions, a coverage reward for the fraction of targets moved in the correct direction above a threshold, a drug_count penalty for parsimony, and a cost penalty for feasibility. The final fitness is a weighted sum of these terms. We search this discrete space with a categorical metaheuristic because the choices are combinatorial, the objective is nonconvex and nondifferentiable due to clipping and thresholds, and gene interactions create many local optima. The metaheuristic treats the objective as a black box, enforces constraints via penalties, and supports efficient add, drop, and swap moves guided by attribution.

In practical terms, the workflow has three steps. First, a LUAD scenario defines which genes should be suppressed or restored. Second, CTD-derived direction-aware drug–gene effects are aligned to this target pattern. Third, the GA searches for small drug sets that best match the target while penalizing redundancy, mismatch, and unnecessarily large combinations.

**Figure 5:**
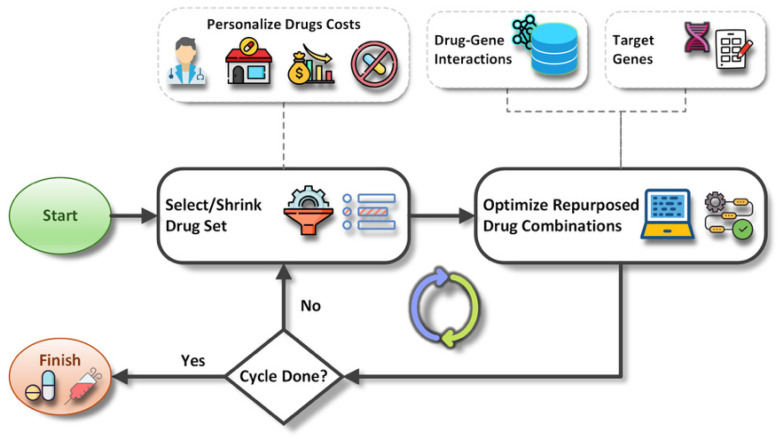
Abstract schema of the proposed method.

Figure 5 illustrates an abstract schema of the cyclic pipeline used in the proposed method. In general, the drugs are selected from a customizable list. This customization may depend on the oncologists’ decisions, drug availability, economic factors, patient restrictions, and other parameters that affect the priority of drug combinations. Each drug is assigned a cost value, which is either positive for avoidance or negative for preference. A cycle ends after optimizing the drug combination sets. The next cycle begins with a reduced list of drugs. This iterative process enables faster filtering of irrelevant or undesired medications in the early stages.

**Figure 6:**
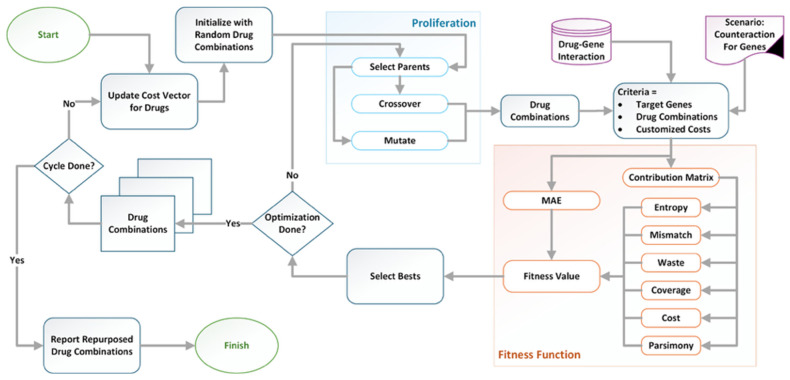
General workflow of the proposed method.

Figure 6 shows the workflow of the proposed method. The pipeline integrates three inputs: scenario-based dysregulated genes with counteraction vectors, drug–gene interactions from CTD, and candidate drug combinations from the GA. First, the model calculates the MAE and a contribution matrix (either proportional or Shapley). Next, it derives contribution-aware terms from this matrix. Then, it forms a weighted fitness by combining MAE with the contribution-aware terms. The GA minimizes this fitness across iterations. After the maximum number of epochs, the algorithm reports the best-fit drug combinations.

All steps were repeated over several cycles. Screening a large set of drugs is difficult for researchers. First, a coarse search was performed across all scenarios for all genes, labelled “Scenario 11”. The allowed combination size was increased to seven. In the next cycle, results from the first cycle were used to keep potent chemicals and remove undesired drugs/chemicals. The literature was reviewed to refine the list. Penalties were applied for toxicity and oncogenic attributes. The remaining drugs/chemicals were passed to the optimizer. The process ended after three cycles in this study. However, additional cycles can be run until a reasonable combination is reached. An alternative setting allows negative costs to favor a chosen set of drugs. This setting corresponds to a search for complementary drugs alongside the preselected agents.

### 3.5. Metaheuristic Optimizer

This study proposes a discrete optimization method based on a GA. The available drugs bound the search space. Each solution is a vector of length *K* (maximum drugs per combination; *K* = 6). Each scenario defines a counteraction vector over genes with desired inhibition (−1) or activation (+1). A candidate *X* encodes a drug combination by drug indices. The genetic algorithm does not guarantee the global optimum, and the chosen fitness weights may influence the ranking of candidate combinations. However, the search space in multi-drug combination design is combinatorially large, making exhaustive evaluation computationally impractical. In such settings, metaheuristic methods such as GA provide a practical way to identify high-quality approximate solutions within a fixed computational budget, defined by a predefined number of iterations and fitness evaluations.

### 3.6. Fitness Function

The combination effect vector is the element-wise sum of the gene-level effects of the selected drugs. The primary objective is to minimize the error between the counteraction vector and the combination effect using MAE. Contribution-aware terms in the fitness function guide the search. These include waste, coverage, drug count, entropy, mismatch, and optional cost. The GA explores, recombines, and mutates combinations to find low-error, well-balanced solutions.

Basic Error Function: The proposed method prefers the MAE (Equation (1)) as the basic fit between the overall drug–effect vector and the target counteraction vector. MAE gives the average absolute deviation per gene, is linear in the residuals, and is easy to interpret. Compared with mean squared error (MSE), MAE is less sensitive to rare large deviations that could distort selection. Unlike the Matthews correlation coefficient (MCC), which requires thresholding and a confusion matrix and discards magnitude, MAE preserves effect size without extra assumptions. For these reasons, MAE is adopted as a robust, transparent core metric that complements the secondary terms.

(1) $$MAE=\frac{1}{G}\sum_{g=1}^{G}\left|c_{g}-{\hat{e}}_{g}\right|$$

where G is the number of genes, $c_{g}$ is the desired counteraction for the gene g, and ${\hat{e}}_{g}$ is the net effect produced by the candidate drug set.

Contribution Matrix: The contribution matrix is computed using either proportional or Shapley attribution, depending on the process’s speed and interaction strength. Proportional attribution is preferred in large inner loops because it is simple, fast, and consistent with an almost-additive superposition. It preserves the sign structure and allocates the net per-gene effect to drugs in proportion to their raw contributions. It is adequate when clipping and gene-level interactions are modest. Shapley attribution is preferred when saturation, redundancy, or synergy are pronounced and a fairness guarantee is needed. It assigns credit by averaging each drug’s marginal contribution across all subset orderings, which respects efficiency, symmetry, the null player, and linearity. In practice, proportional attribution can be used during search, and Shapley attribution can be used to audit top solutions or finalize reports when higher fidelity is required.

Let $r_{g,j}$ be drug j’s raw signed effect on the gene g, S the selected drugs, and ${\hat{e}}_{g}=clip(\sum_{k\in S}r_{g,k},-1,1)$ with $S_{g}=\sum_{k\in S}r_{g,k}\neq 0$ proportional attribution sets the contribution using Equation (2) so that $\sum_{j\in S}a_{g,j}={\hat{e}}_{g}$.

(2) $$a_{g,j}={\hat{e}}_{g}\frac{r_{g,j}}{S_{g}},\;and\;a_{g,j}=0\;if\;s_{g}=0$$

For Shapley attribution, define $f_{g}\left(X\right)=clip(\sum_{k\in X}r_{g,k},-1,1)$ for any subset T⊆S. The Shapley value of the drug j for gene g is calculated by Equation (3), typically estimated by Monte Carlo permutations in practice.

(3) $$\emptyset_{g,j}=\sum_{T\subseteq S\;|\;\{j\}}\frac{\left|T\right|!\left(\left|S\right|-\left|T\right|-1\right)!}{\left|S\right|!}[f_{g}\left(T\cup \left\{j\right\}\right)-f_{g}(T)]$$

Redundancy (Wasted Same-Sign) Penalty: This term punishes overshooting the target in the correct direction. For each gene i, we added up the credited magnitudes from drugs that push in the same direction as the counteraction $C_{i}$, and compared that total to the desired magnitude $\left|C_{i}\right|$. Any excess beyond $\left|C_{i}\right|$ is counted as “waste”. It is symmetric for up- and downregulation because it uses magnitudes, and it complements MAE. MAE penalizes being short of the target, while this penalty activates only when the right direction is overfilled.

(4) $$R_{waste}\left(x\right)=\sum_{i}max(0,\;\sum_{j:sgn\left(a_{ij}\right)=sgn(C_{i})}\left|a_{ij}\right|-\left|C_{i}\right|)$$

Mismatch Penalty: This term penalizes credited effects that oppose the target direction. For gene i, any attribution $a_{ij}$ with a sign different from $C_{i}$ counts as a mismatch. This penalty, as formulated in Equation (5), targets directional inconsistency, rather than magnitude errors.

(5) $$R_{mis}\left(x\right)=\sum_{i}\;\sum_{j:sgn\left(a_{ij}\right)\neq sgn(C_{i})}\left|a_{ij}\right|$$

Entropy (Per Gene) Penalty: This term penalizes the dispersion of credited effects across drugs. Equation (6) denotes the probability-like weight for the gene i, and Equation (7) calculates its entropy. The overall entropy of the drug combination is given by Equation (8).

(6) $$p_{ij}=\frac{\left|a_{ij}\right|\;1\;\left[sgn\left(a_{ij}\right)=sgn(C_{i})\right]}{\sum_{k}\left|a_{ik}\right|\;1\;\left[sgn\left(a_{ik}\right)=sgn(C_{i})\right]}$$

(7) $$H_{i}=-\sum_{j}p_{ij}\;log(p_{ij})$$

(8) $$R_{entropy}(x)=\sum_{i}H_{i}$$

Coverage Reward: Equation (9) denotes the rewards that meet the target magnitude in the correct direction for genes. The overall reward aggregates Cov across genes (optionally weighted). It favors direction-correct fulfillment of targets while not encouraging overshoot.

(9) $$Cov=\sum_{i}\omega_{i}\frac{min(\left|C_{i}\right|,\;\sum_{j:sgn\left(a_{ij}\right)=sgn(C_{i})}\left|a_{ij}\right|}{\left|C_{i}\right|}$$

Drug-Level Parsimony/Cost: This term discourages the use of large regimens and accounts for reducing undesired parameters, such as budget, toxicity, and other customized motivations. Indeed, it helps to exclude certain drugs that may not be suitable for personalized medicine. Equation (10) denotes the credited usage of drug j. A parsimony penalty function counts the used drugs utilizing Equation (11). Additionally, the cost assigned for drug usage is calculated using Equation (12). This study ignores the cost by setting the corresponding vector to all zeros at Cycle 1.

(10) $$\phi_{j}=\sum_{i}\left|a_{ij}\right|$$

(11) $$R_{count}=\sum_{j}1(\phi_{j}>\varepsilon )$$

(12) $$R_{cost}=\sum_{j}c_{j}(\phi_{j}>\varepsilon )$$

Fitness Value: Finally, this study minimizes a composite loss that balances accuracy with contribution-aware structure, as defined in Equation (13).

(13) $$J=\alpha MAE+\beta R_{waste}+\gamma R_{mis}+\eta R_{entropy}+\lambda R_{count}+\kappa R_{cost}-\tau Cov$$

where weights α, β, γ, η, λ, κ, τ≥0 control trade-offs and normalize each term (e.g., by gene count or a baseline maximum) before tuning.

Many drug sets can achieve the same error by canceling opposing effects or by overshooting and then clipping. While the mismatch term removes directionally wrong credit, the redundancy term prevents same-sign overshoot that wastes effect under saturation. At the same time, the entropy term discourages diffuse, redundant credit, and promotes parsimonious, interpretable regimens. Drug-level parsimony and cost align the search with clinical feasibility and budgetary and toxicity constraints. The coverage reward explicitly favors meeting targets in the correct direction rather than relying on cancellations. These terms break MAE ties, reduce weak solutions, and tend to improve out-of-sample robustness and translational plausibility.

### 3.7. Parameterization

The optimization was implemented using a GA. The hyperparameters were a crossover rate of 0.9 and a mutation rate of 0.2. Additionally, this study employed three consecutive cycles to reduce the number of drugs and enhance the relevance of the suggested combinations. The first cycle (coarse search) used a population of 500 with 100 iterations. The second cycle used 250 agents with 70 iterations. The final cycle (fine search) used 150 agents with 10 iterations. To improve robustness, the GA was run 10 times per scenario. The search space allowed drug combinations of up to six drugs. The fitness function prioritized MAE with α = 1. Secondary terms used *β* = 0.2 for waste, *γ* = 0.2 for mismatch, and *τ* = 0.2 for coverage. Lower weights were used for entropy (*η* = 0.05) and drug count (*λ* = 0.05). No cost term was applied (*κ* = 0) for Cycle 1. For the next cycles, a cost coefficient (*κ* = 1) was set. The process block was executed twice, once with proportional contributions and once with Shapley contributions. For Shapley, 256 permutations were sampled. These coefficients were fixed a priori and kept constant across scenarios to provide a transparent proof-of-concept setting; they should therefore be interpreted as design choices rather than uniquely optimal values.

### 3.8. Orthogonal Computational Validation

An orthogonal computational validation step was applied to the prioritized drug candidates using CLUE/Connectivity Map transcriptomic perturbation results. This step was designed as an external support layer and was not used to retrain, optimize, or redefine the primary prediction framework. A signed LUAD disease query was used, with separate upregulated and downregulated gene sets (Scenario 11). The resulting CLUE connectivity output was interpreted in terms of transcriptomic reversal. Negative connectivity values were treated as supportive because they indicated reversal of the disease-associated signature, whereas positive values were treated as unsupportive because they indicated similarity to the disease signature.

For downloaded result tables, the main score column was taken from the available CLUE output field, such as TAG when present. Drug ranking was based on a transparent rule-based procedure. Lung and lung cancer cell lines were prioritized over other cellular contexts. Rows with qc_pass = 1 and is_ncs_sig = 1 were given priority, and additional weight was assigned to is_hiq = 1. More negative scores were rewarded, consistent negative results across high-quality lung rows were favored, and strong positive scores in high-quality lung rows were penalized. Evidence from non-lung cell lines was considered only as secondary context. For drug combinations, only component-level orthogonal support was considered unless direct combination perturbation data were available. This validation layer was used to provide external transcriptomic reversal support and did not replace experimental validation, clinical assessment, or toxicity evaluation.

## 4. Conclusions

This work introduces a contribution-aware framework to search for rational multi-drug combinations for LUAD. Relative to our Biomolecules 2025 review [9], the novelty of this study lies not in re-reviewing LUAD biology or repurposing methods, but in proposing and applying a contribution-aware computational framework for scenario-based small drug-combination prioritization. To specify which genes should be down- or upregulated, we defined ten LUAD scenarios from the current literature and encoded them as gene-level counteraction vectors. Using direction-aware drug–gene interactions from CTD, we built a contribution matrix. We applied a GA to identify small combinations that best matched these counteraction vectors under multiple constraints.

Results show that while larger regimens offer only modest gains, increasing the combination size from 1 to 3 drugs usually yields a clear reduction in MAE. Scenarios with many dysregulated genes or mixed directions require richer combinations to achieve good fits. Indeed, focused modules can often be counteracted with only 2 or 3 drugs. Clustering of drugs and genes reveals groups of interchangeable candidates and shared mechanisms across scenarios, such as agents that repeatedly appear as alternatives around the same gene clusters. The compound-level literature review supports the biological plausibility of several candidates, although their translational maturity ranges from clinically used drugs to exploratory preclinical agents.

Methodologically, the framework makes two contributions. First, scenario-based counteraction vectors provide a transparent bridge between driver-focused narratives and a computational search space. These scenarios can be replaced in practice with patient-specific profiles. Second, the contribution-aware fitness function works at the per-drug, per-gene level and balances the desired counteraction with waste, mismatch, entropy, coverage, combination size, and optional cost. This design is intended to address the limitations of unweighted signature-matching and single-score synergy-screening approaches while making the resulting combinations more interpretable and easier to audit.

### 4.1. Limitations

This study has several important limitations that should be considered when interpreting the results.

First, our framework relies heavily on drug–gene interactions curated in the Comparative Toxicogenomics Database. These data are incomplete and biased toward well-studied drugs, genes, and pathways. We also aggregated direction labels across different cell types, doses, and diseases. As a result, the estimated up- or downregulation for a given drug–gene pair may not fully reflect its effect in LUAD cells or clinical conditions.

Second, the ten LUAD “scenarios” and their counteraction vectors were built from the literature and expert curation, not from individual patients. The gene lists are necessarily incomplete and reflect current knowledge about common drivers and pathways. Rare drivers, intratumoral heterogeneity, and non-genetic mechanisms are underrepresented. We also model the desired change for each gene as a simple +1 or −1, which ignores the strength and nonlinearity of biological responses.

Third, our model treats each drug as a fixed vector of gene-level effects and does not include dose, pharmacokinetics, pharmacodynamics, or scheduling. We do not model drug–drug interactions, target engagement, metabolism, or tissue distribution. In practice, the safety and efficacy of any combination depend strongly on dose and exposure, which were outside the scope of our current optimization. Some components of the candidate combinations are tool compounds, herbal extracts, or drugs with limited oncology data, and we did not incorporate formal toxicity scores or clinical interaction data.

Fourth, the GA is a heuristic search method and cannot guarantee that the identified combinations are globally optimal. The objective function and its weights were chosen based on reasonable, but subjective, design decisions. Different choices of weights or penalty terms could favor different solutions. We also limited the maximum combination size, which restricts the explored space and may miss larger but potentially useful regimens. Therefore, systematic testing of alternative weight configurations and sensitivity analysis of these choices are important directions for future work.

Fifth, this work is purely in silico. We do not provide experimental validation of the predicted combinations in LUAD cell lines, organoids, or animal models. We provide only orthogonal computational support via CLUE/CMap transcriptomic reversal analysis and did not perform systematic validation in independent pharmacogenomic response datasets. Therefore, all suggested combinations should be considered hypothesis-generating only and not as treatment recommendations.

Finally, the current framework is tuned to LUAD and to the specific set of curated scenarios. Its direct generalization to other cancer types, to non-cancer diseases, or to fully patient-specific profiles will require additional development. In particular, integrating multi-omics data, incorporating toxicity and interaction models, and adding experimental feedback loops are essential next steps before any clinical translation.

### 4.2. Future Directions

This study is purely in silico and should be viewed as hypothesis-generating. The current implementation does not model dose, pharmacokinetics, pharmacodynamics, or toxicity, and it relies on curated CTD interactions and literature-based scenarios. Future work should integrate patient-derived multi-omics data, pharmacogenomic response resources, and toxicity or interaction models. Experimental testing of a small, carefully selected subset of combinations in LUAD cell lines, organoids, or in vivo models will be essential. With these extensions, the proposed framework may support more systematic and transparent design of repurposed drug combinations for LUAD and other cancers.

## Figures and Tables

**Table 1:** Summary of LUAD scenarios used in the contribution-aware optimization framework.

Scenario	Biological Axis	Representative Genes in Module	Desired Counteraction
1	RTK activation	*EGFR*, *ERBB2*, *MET*, *ALK*, *ROS1*	Suppress activated RTK signaling
2	*EGFR* exon 20 insertion	*EGFR*, *ERBB2*, *ERBB3*	Suppress ERBB signaling in exon 20-driven tumors
3	*KRAS* pathway activation	*KRAS*, *PTPN11*, *SOS1*, *MAP2K1*, *MAP2K2*, *MAPK1*, *MAPK3*	Suppress *KRAS*–MAPK signaling
4	*STK11*/*KEAP1* co-alteration	*STK11*, *PRKAA1*, *PRKAA2*, *KEAP1*, *NFE2L2*, *GLS*	Restore *STK11*/AMPK and *KEAP1*; suppress NRF2/*GLS*-associated adaptation
5	*BRAF*–MAPK activation	*BRAF*, *MAP2K1*, *MAP2K2*, *MAPK1*, *MAPK3*	Suppress *BRAF*–MAPK signaling
6	PI3K/AKT/mTOR activation	*PIK3CA*, *AKT1*, *AKT2*, *AKT3*, *MTOR*, *RPS6KB1*, *PTEN*	Suppress PI3K/AKT/mTOR; restore *PTEN*
7	*ERBB3*–NRG1 activation	*ERBB3*, *ERBB2*, NRG1, *PIK3CA*	Suppress HER3-centered escape signaling
8	DDR dysfunction with *ATR* dependency	*ATR*, *CHEK1*, *WEE1*, *PARP1*, *ATM*, *BRCA1*, *BRCA2*	Suppress checkpoint rescue; restore major DDR functions
9	*RET*/NTRK fusions	*RET*, *NTRK1*, *NTRK2*, *NTRK3*, *MAP2K1*, *MAP2K2*	Suppress fusion-driven kinase signaling
10	*SMARCA4*-deficient LUAD	*SMARCA4*, *SMARCA2*, *EZH2*, *CDK4*, *CDK6*	Restore *SMARCA4*-related function; suppress compensatory dependencies

**Table 2:** Clusters of dysregulated genes and corresponding drug clusters across scenarios.

Scenario	Reg	Gene Cluster	Drug Alternatives	Group
1	Down	*ROS1*	I. jinfukang	A
	Down	*ERBB2*, *ALK*, *MET*, *EGFR*	I. tanespimycin	B
2	Down	*ERBB2* *EGFR*	bisdemethoxycurcumin tanespimycin	A
	Down	*ERBB3*	I. triphenyl(phenylethynyl)phosphonium	B
3	Down	*MAP2K2*, *MAP2K1*, *PTPN11*, *KRAS*, *MAPK1*	I. ivermectin	A
	Down	*MAPK3*, *SOS1*, *MAPK1*	I. bisdemethoxycurcumin	B
4	Down	*NFE2L2*	I. fenofibrate	A
	Up	*PRKAA1*, *STK11*		
	Down	*GLS*	zinc acetate tanespimycin motexafin gadolinium metformin	B
	Up	*KEAP1*	I. silymarin	C
	Up	*PRKAA2*	I. telmisartan	D
5	Down	*MAPK1*, *MAPK3*	3,5-dihydroxy-4-methoxybenzyl alcohol asparanin A bisdemethoxycurcumin oleanolic acid	A
	Down	*MAP2K2*, *MAP2K1*, *MAPK1*	I. ivermectin	B
	Down	*BRAF*	I. triphenyl(phenylethynyl)phosphonium	C
6	Down	*MTOR*, *PIK3CA*, *AKT1*, *RPS6KB1*	I. ivermectin	A
	Down	*AKT3*	I. peracetylated N-azidoacetylmannosamine	B
	Up	*PTEN*	KR-62980 allyl isothiocyanate DLBS 1425 2-chloro-5-nitrobenzanilide	C
7	Down	*PIK3CA*	3,5-dihydroxy-4-methoxybenzyl alcohol ivermectin atorvastatin asparanin A metformin alisol B 23-acetate	A
	Down	*ERBB2*	napabucasin SR 144528 bisdemethoxycurcumin tanespimycin tetrandrine cordycepin	B
	Down	*ERBB3*	I. triphenyl(phenylethynyl)phosphonium	C
8	Down	*PARP1*	cladribine solasodine 1,2,5,8-tetrahydroxy anthraquinone HS 1200	A
	Down	*WEE1*, *CHEK1*	I. thapsigargin	B
	Up	*BRCA2*, *BRCA1*		
	Down	*ATR*	zinc acetate motexafin gadolinium	C
	Up	*ATM*		
9	Down	*RET*	I. thapsigargin	A
	Down	*NTRK2*	staurosporine aglycone terpenes	B
	Down	*MAP2K2*	tetrahydropalmatine vanillin	C
10	Down	*EZH2*	piroxicam enzalutamide	A
	Down	*CDK4*, *CDK6*	dieckol GSK1210151A NVP-AEW541 panduratin A cannabidiol HS 1200	B
	Up	*SMARCA4*	I. darinaparsin	C

**Table 3:** Compound CLUE match.

CLUE Support Category Count	Compounds	Basis of Support
Strong 14	Direct CLUE match: Atorvastatin, Cladribine, Darinaparsin, Enzalutamide, Ivermectin, Metformin, NVP-AEW541, Piroxicam, Tanespimycin, Telmisartan, Thapsigargin. Mapped representative: Bisdemethoxycurcumin (curcumin), Staurosporine aglycone (staurosporine), Terpenes (betulinic acid).	Strong orthogonal transcriptomic reversal support was observed either from direct CLUE perturbagen matches or from closely related mapped representatives.
Moderate 6	Direct CLUE match: Cordycepin, Fenofibrate, Oleanolic Acid, SR 144528, Tetrahydropalmatine. Mapped representative: 2-Chloro-5-nitrobenzanilide (GW-9662).	Moderate orthogonal transcriptomic reversal support was observed, although the signal was less strong or less consistent than in the strong-support group.
Weak 1	Tetrandrine	Limited negative transcriptomic reversal support was observed under the current ranking framework.
Not supported 21	No usable direct CLUE match under current mapping: KR-62980, DHMBA, Alisol B 23-acetate, Allyl isothiocyanate, Asparanin A, Cannabidiol, Dieckol, GSK1210151A, HS 1200, Motexafin gadolinium, Napabucasin, Solasodine, Triphenyl(phenylethynyl)phosphonium, Vanillin. Extract/mixture/formulation-level entities: DLBS 1425, Jinfukang, Silymarin. Candidate equivalent existed but no usable filtered support was retained: Panduratin A, Ac4ManNAz, Zinc Acetate. Related representative only: 1,2,5,8-Tetrahydroxy anthraquinone.	No usable orthogonal CLUE-based support was assigned under the current mapping and filtering framework; this does not necessarily indicate a lack of biological activity.

## Data Availability

The original contributions presented in this study are included in the article/supplementary material. Further inquiries can be directed to the corresponding author.

## Supplementary Materials

The following supporting information can be downloaded at: Supplementary File S1: (1_iarc_agents.csv) https://github.com/sajjad-nematzadeh/DrugRepurposingLUAD/blob/main/1_iarc_agents.csv (accessed on 25 March 2026). Supplementary File S2: (2_ntp_roc.csv) https://github.com/sajjad-nematzadeh/DrugRepurposingLUAD/blob/main/2_ntp_roc.csv (accessed on 25 March 2026). Supplementary File S3: (3_PAN.xlsx) https://github.com/sajjad-nematzadeh/DrugRepurposingLUAD/blob/main/3_PAN.xlsx (accessed on 25 March 2026). Supplementary File S4: (4_chemicals_pubchem.csv) https://github.com/sajjad-nematzadeh/DrugRepurposingLUAD/blob/main/4_chemicals_pubchem.csv (accessed on 25 March 2026). Supplementary File S5: (5_cycle2.xlsx) https://github.com/sajjad-nematzadeh/DrugRepurposingLUAD/blob/main/5_cycle2.xlsx (accessed on 25 March 2026). Supplementary File S6: (orth_val_clue.xlsx) https://github.com/sajjad-nematzadeh/DrugRepurposingLUAD/blob/main/orth_val_clue.xlsx (accessed on 25 March 2026).

## Appendix A

This study includes a Scenarios section to connect core LUAD biology with practical repurposing. Each scenario captures a recurrent mechanism that changes prognosis or treatment response. We selected ten major disease axes from the literature. These covered receptor activation, RAS–MAPK and PI3K signaling, fusion kinases, immune-cold genotypes, DNA damage repair, and chromatin remodeling. Selection criteria were prevalence in LUAD, clinical actionability, clear downstream pathways, known or plausible druggable nodes, resistance relevance, and cross-cancer transferability [9].

All scenarios used one uniform template for clarity and comparison. We begin with a brief description that outlines the clinical and biological context. Next, key genes are outlined, along with their specific dysregulations. Moreover, the affected functions and pathways are described, and the most relevant current therapeutic counteractions are summarized. Additionally, each scenario includes a counteraction section that illustrates the desired regulation. The plus (+) signs represent the desired increase or restoration of the expression. On the other hand, minus (−) is used for a decrease expression or an inhibition target. We close with cross-cancer relevance to support repurposing logic. In the Methods section, a metaheuristic algorithm searches for drug–gene interaction resources and computes repurposed drug groups for each scenario.

### Appendix A.1. Scenario 1. Receptor Tyrosine Kinase (RTK)

RTKs are major drivers in LUAD. Alterations include mutations, exon skipping, fusions, and amplification. These changes, which activate growth and survival pathways, create therapeutic targets. Recent reviews summarize mechanisms and clinical impact across cancers [32].

Genes and dysregulation: “Epidermal Growth Factor Receptor” (EGFR)-activating mutations increase pathway output and may co-occur with gene amplification. “*MET* exon 14 skipping-type mutation” (METex14) stabilizes the *MET* proto-oncogene and sustains its signaling. “Anaplastic Lymphoma Kinase” (*ALK*) and *ROS1* fusions create kinase activity. “Human Epidermal growth factor Receptor 2” (HER2) amplification and exon 20 insertions define a subgroup with actionable mutations in NSCLC. Key clinical datasets include capmatinib in METex14, lorlatinib over crizotinib in *ALK*, and repotrectinib in *ROS1*. Recent studies outline HER2-altered NSCLC care [33,34,35,36].

Affected functions and pathways: Dysregulated RTKs drive “Mitogen-Activated Protein Kinase” (MAPK)/“Extracellular signal-Regulated Kinase” (ERK), “Phosphoinositide 3-Kinase” (PI3K)/AKT/“mechanistic Target Of Rapamycin” (mTOR), and “Janus Kinase” (JAK)/“Signal Transducer And Activator of Transcription” (STAT) signaling pathways. The result is proliferation, survival, invasion, and angiogenesis [32].

Candidate therapeutic counteractions: Classical *EGFR* mutations are responsive to third-generation “Tyrosine Kinase Inhibitors” (TKIs) such as osimertinib. METex14 tumors respond to capmatinib or tepotinib. *ALK*-positive disease shows durable benefit with first-line lorlatinib. *ROS1*-positive disease benefits from repotrectinib, which exhibits strong central nervous system (CNS) activity. HER2-altered NSCLC responds to trastuzumab deruxtecan and other HER2-directed agents [33,34,35,36].

Counteraction vector: *EGFR*-, *ERBB2*-, *MET*-, *ALK*-, *ROS1*- [34].

Cross-cancer relevance: RTK alterations recur in breast, gastric, colorectal, and other cancers [32,36].

### Appendix A.2. Scenario 2. EGFR Exon 20 Insertion

*EGFR* ex20 insertions define a distinct LUAD subgroup. These mutations are resistant to earlier EGFR TKIs and require suitable strategies. The PAPILLON Phase 3 trial established amivantamab plus chemotherapy as a first-line option [37]. Contemporary reviews explain clinical implications and testing needs [38].

Genes and dysregulation: Exon 20 insertions are in-frame additions near the αC-helix and the following loop. They stabilize the active conformation and prevent binding by earlier TKIs. One work defines pocket changes that guide selective inhibitor design [39].

Affected functions and pathways: Constitutive EGFR signaling activates the MAPK/ERK and PI3K/AKT pathways, which support persistent growth and survival. These features explain weaker responses to earlier TKIs [38].

Candidate therapeutic counteractions: First-line amivantamab with carboplatin and pemetrexed improves progression-free survival compared to chemotherapy alone [37]. After platinum therapy, sunvozertinib shows activity with a manageable safety profile [40]. Zipalertinib provides additional benefit [41].

Counteraction vector: *EGFR*-, *ERBB2*-, *ERBB3*- [37].

Cross-cancer relevance: The exon 20 insertion concept extends to *ERBB2*. Pan-cancer studies reveal shared activation mechanisms and inform the design of selective agents [42]. Additionally, *EGFR* exon 20 insertions are rare in gliomas [43].

### Appendix A.3. Scenario 3. KRAS Pathway Activation

*KRAS* activation is a frequent and central driver in LUAD. Two randomized trials now define the activity of direct *KRAS* G12C inhibition in previously treated disease. Sotorasib improved progression-free survival over docetaxel. Adagrasib was later shown to provide greater benefit and a stronger response. These data establish *KRAS* pathway blockade as a validated therapeutic axis in LUAD [44,45].

Genes and dysregulation: Recurrent missense mutations at codons 12 and 13 alter *KRAS* cycling and keep RAS in an active signaling state. G12C is druggable with covalent inhibitors, whereas other alleles, such as G12D and G12V, remain challenging to target with this approach. Resistance arises through pathway reactivation, co-alterations, and secondary *KRAS* variants [46,47].

Affected functions and pathways: Mutant *KRAS* drives RAF–MEK–ERK signaling and fuels feedback through RTK signaling. An upstream dependency creates an SHP2-mediated bypass that restores MAPK flux after *KRAS* blockade. Dual *KRAS* and SHP2 inhibition restores suppression and improves antitumor immunity in preclinical models [48].

Candidate therapeutic counteractions: For patients with *KRAS* G12C, a covalent inhibitor is used after chemo-immunotherapy. Adagrasib improved progression-free survival and response rates compared to docetaxel in a Phase 3 trial. Sotorasib also outperformed docetaxel in terms of progression-free survival. These rational combinations involve adaptive signaling, including SHP2 co-inhibition in trials [44,45,48].

Counteraction vector: *KRAS*-, *PTPN11*-, *SOS1*-, *MAP2K1*-, *MAP2K2*-, *MAPK1*-, *MAPK3*- [45].

Cross-cancer relevance: *KRAS* is a central oncogenic node in both colorectal and pancreatic cancers. Lessons from allele-specific and emerging pan-*KRAS* approaches inform drug-resistance management and combination design in LUAD [47,49].

### Appendix A.4. Scenario 4. STK11 and KEAP1 Co-Alterations

Alterations in “Serine/Threonine Kinase” (*STK11*) and “Kelch-Like ECH-Associated Protein 1” (*KEAP1*) define an immune-cold LUAD subset with inferior outcomes on “Programmed cell Death 1” (PD-1) or “Programmed cell Death-Ligand 1” (PD-L1) therapy. Recent clinical-genomic analyses show limited benefit from chemo-immunotherapy alone and support adding “Cytotoxic T-Lymphocyte-Associated protein 4” (CTLA-4) blockade to restore activity [50,51].

Genes and dysregulation: *STK11* loss turns off “Liver Kinase B1” (LKB1)-“AMP-Activated Protein Kinase” (AMPK) signaling and promotes metabolic adaptation. *KEAP1* loss stabilizes NRF2 and rewires redox control, glutamine use, and stress responses. Together, they suppress antigen presentation and prevent T cell activation. New works show druggable control points within the NRF2 stability machinery [52,53].

Affected functions and pathways: This genotype reduces interferon signaling, lowers PD-L1 expression in *STK11*-mutant tumors, and drives a myeloid-skewed inflammatory response. It blunts the response to PD-L1 and dampens the benefit of adding PD-L1 to chemotherapy. Adding CTLA-4 to chemo-immunotherapy can overcome part of this resistance and lengthen survival in this subgroup [50].

Candidate therapeutic counteractions: Combination immunotherapy with CTLA-4 and a platinum doublet can be prioritized when *STK11* or *KEAP1* mutations are present. Pairing *KRAS*-targeted agents for co-mutant disease should also be enriched. In addition, testing redox or glutamine-axis strategies in NRF2-high tumors can be beneficial. Preclinical data support degrading or destabilizing NRF2, which cooperates with glutaminase inhibition to reduce tumor growth [50,53].

Counteraction vector: *STK11*+, *PRKAA1*+, *PRKAA2*+, *KEAP1*+, *NFE2L2*-, *GLS*- [50].

Cross-cancer relevance: *KEAP1*-NRF2 dysregulation appears across squamous lung, head and neck, esophageal, and liver cancers. The same metabolic stress programs and immune evasion patterns recur, which supports pathway-directed strategies beyond LUAD [52].

### Appendix A.5. Scenario 5. BRAF-MAPK Pathway Activation

A subset of LUAD carries activating *BRAF* mutations. These tumors depend on MAPK signaling. They respond to combined *BRAF* and MEK inhibition in carefully selected settings. Clinical data support this strategy in BRAF^V600E^ (valine at codon 600 is replaced by glutamic acid) disease [54,55].

Genes and dysregulation: *BRAF* mutations occur in about 3–5% of NSCLC cases. Class 1 mutations are V600 and signal as monomers. Classes 2 and 3 are non-V600 dimers with distinct biology and lower sensitivity to targeted therapy [54,55].

Affected functions and pathways: Mutant *BRAF* drives persistent MEK–ERK signaling, which promotes proliferation and survival. MAPK feedback can reactivate ERK and cause resistance if *BRAF* is blocked alone. Dual BRAF–MEK blockade reduces this escape [54,55].

Candidate therapeutic counteractions: Dabrafenib plus trametinib improves outcomes in BRAF^V600E^ NSCLC. Recent data confirm activity and manageable safety in diverse cohorts. Encorafenib plus binimetinib is also active and may be considered where available. Choice can reflect toxicity profiles and access [55,56].

Counteraction vector: *BRAF*-, *MAP2K1*-, *MAP2K2*-, *MAPK1*-, *MAPK3*- [56].

Cross-cancer relevance: BRAF^V600E^ is an established driver in melanoma and other tumors. Tumor-agnostic approvals for *BRAF*–MEK combinations inform lung cancer strategy. Lessons on sequencing with immunotherapy come from melanoma and may translate with care [55,57].

### Appendix A.6. Scenario 6. PI3K/AKT/mTOR Axis Activation

The PI3K/AKT/mTOR axis is a central pathway in the growth of LUAD. It contributes to tumor progression and to therapy resistance. Recent reviews highlight its clinical relevance and therapeutic opportunities [58,59].

Genes and dysregulation: Hotspot *PIK3CA* mutations cluster at E545K, E542K, and H1047R. They often co-occur with alterations in *EGFR*, *KRAS*, or “Tumor Protein p53” (TP53). *AKT1* E17K (at codon 17 of the *AKT1* protein, glutamate is replaced by lysine) is an activating hotspot at a lower frequency. *PTEN* loss removes a key brake on PI3K signaling [59,60,61].

Affected functions and pathways: PI3K activation promotes cell survival, metabolism, and growth. It interacts with MAPK signaling and the tumor microenvironment. Axis activation can blunt immune responses and support persistence under pressure from targeted therapy [58,59].

Candidate therapeutic counteractions: Direct inhibition of PI3K, AKT, or mTOR in NSCLC is currently under active investigation. Early-phase trials of dual mTORC1/mTORC2 blockade show feasibility with biologic activity in pathway-altered tumors. Taselisib did not work well enough in the LUNG-MAP trial arm, so it was stopped early. This shows that selecting the right biomarkers and using carefully chosen drug combinations are crucial. In *EGFR*-mutant NSCLC with acquired *PIK3CA* mutations, combined pathway targeting is a rational approach in trials [62,63,64].

Counteraction vector: *PIK3CA*-, *AKT1*-, *AKT2*-, *AKT3*-, *MTOR*-, *RPS6KB1*-, *PTEN*+ [65].

Cross-cancer relevance: Inhibition of the AKT pathway has shown clinical benefit in other cancers. Capivasertib plus fulvestrant improved progression-free survival and gained regulatory approvals in biomarker-selected breast cancer [65].

### Appendix A.7. Scenario 7. ERBB3-NRG1 Pathway Activation and HER3-Directed ADCs

HER3 becomes an adaptive escape node after *EGFR* TKI therapy in LUAD. In addition, oncogenic NRG1 fusions can also activate it. These patterns are now actionable with HER3-directed antibody drug conjugates and with a HER2×HER3 bispecific antibody in NRG1-rearranged disease [66]. In a report, HER3-DXd showed antitumor activity with translational correlates in *EGFR*-mutant NSCLC after osimertinib and chemotherapy [67]. The eNRGy trial phase II study confirmed the efficacy of zenocutuzumab for NRG1-fusion cancers, including NSCLC [68].

Genes and dysregulation: *ERBB3* (HER3) is frequently upregulated in *EGFR*-mutant tumors at the time of progression. NRG1 fusions create paracrine or autocrine HER3 ligands that drive HER2–HER3 dimerization. These events reactivate PI3K–AKT and MAPK signaling despite prior kinase inhibition [66]. The NEJM eNRGy study establishes NRG1 fusions as recurrent drivers across solid tumors and documents the prevalence of NSCLC within the cohort [68].

Affected functions and pathways: Ligand-driven or receptor-level activation of HER3 restores downstream survival signaling. The main axes are PI3K–AKT and RAS–MAPK. This signaling is sufficient to sustain growth under *EGFR* blockade. It also creates drug-tolerant states that enable heterogeneous resistance [66].

Candidate therapeutic counteractions: Patritumab deruxtecan (HER3-DXd) delivers a topoisomerase-I payload to HER3-expressing cells. Early- and expansion-cohorts in *EGFR*-mutant NSCLC showed objective responses and clinically meaningful overall survival with *EGFR* TKI and platinum chemotherapy [66]. One analysis reports the extended activity and biomarker findings for HER3-DXd in this setting [67]. For NRG1 fusions, zenocutuzumab produced durable responses across tumor types [68].

Counteraction vector: *ERBB3*-, *ERBB2*-, NRG1-, *PIK3CA*- [68].

Cross-cancer relevance: NRG1 fusions occur in multiple cancers. The eNRGy trial showed efficacy in pancreatic cancer and NSCLC [68].

### Appendix A.8. Scenario 8. DNA Damage Response Dysfunction (DDR) with ATR Dependency

Alterations in the DDR pathway are common in LUAD. They shape genomic instability and treatment response. Recent clinical and translational work highlights *ATR* dependency and supports the rational combination of immunotherapy and “Poly ADP-Ribose Polymerase” (PARP) blockade in biomarker-selected patients [69]. In a paper, durvalumab plus the *ATR* inhibitor ceralasertib showed the strongest benefit among biomarker-guided modules in advanced NSCLC [70].

Genes and dysregulation: Key events include loss or mutation of *ATM*, *BRCA1*, *BRCA2*, and TP53. These lesions disrupt homologous recombination and checkpoint control. Furthermore, they increase replication stress and reliance on *ATR* signaling for survival [69,70]. Clinical biomarker cohorts suggest that *ATM*-altered tumors can be sensitized to *ATR* inhibition in combination with PD-L1 blockade [70].

Affected functions and pathways: DDR defects impair double-strand break repair and fork protection, shifting cells toward *ATR*-CHK1 signaling to manage replication stress. As a result, this procedure creates a therapeutic liability. Preclinical data show that intermittent *ATR* blockade can prime antitumor immunity and enhance anti-PD-L1 activity through type I interferon signaling [71].

Candidate therapeutic counteractions: *ATR* inhibitors such as ceralasertib have shown clinical activity across tumors with genomic instability. A study reported durable benefit with ceralasertib, including signals in ARID1A-deficient and DDR-defective cancers [72]. In NSCLC, durvalumab plus ceralasertib produced higher response and longer survival than other modules in the HUDSON platform. The *ATM*-altered cohort showed the greatest effect size [70]. PARP inhibitors remain an option in selected “Homologous Recombination” (HR)-deficient contexts and are under investigation in NSCLC, both alone and in combination with *ATR* inhibition [69].

Counteraction vector: *ATR*-, *CHEK1*-, *WEE1*-, *PARP1*-, *ATM*+, *BRCA1*+, *BRCA2*+ [70].

Cross-cancer relevance: *ATR* inhibition has produced clinical signals in diverse solid tumors [72].

### Appendix A.9. Scenario 9. RET and NTRK Kinase Fusions

*RET* and NTRK fusions define actionable LUAD. Selpercatinib improved first-line outcomes versus chemotherapy. It also showed protection against the development of new brain metastases. Final trial reports confirm durable efficacy. Testing should include RNA-based methods to detect rare NTRK fusions [73,74,75,76].

Genes and dysregulation: *RET* fusions, such as KIF5B-*RET*, create a constitutively active kinase. *NTRK1*, *NTRK2*, and *NTRK3* fusions form ligand-independent TRK signaling. These events activate growth pathways and can present with early brain spread. In short, accurate fusion calling benefits from RNA-based next-generation sequencing [74,76].

Affected functions and pathways: *RET* and TRK fusions activate the MAPK and PI3K signaling pathways. They promote proliferation, survival, and tropism in the central nervous system. Brain-penetrant inhibition limits intracranial progression in first-line *RET*-positive disease [74].

Candidate therapeutic counteractions: Selpercatinib is preferred for *RET*-positive NSCLC. It improved progression-free survival and showed strong intracranial activity. Pralsetinib also has clinical activity in both trials and real-world cohorts. For NTRK fusions, larotrectinib and entrectinib are standards with durable benefit across tumors, including lung cancer [73,76,77].

Counteraction Vector: *RET*-, *NTRK1*-, *NTRK2*-, *NTRK3*-, *MAP2K1*-, *MAP2K2*- [73].

Cross-cancer relevance: TRK fusions are observed across various cancers. *RET* fusions also appear beyond the lung [76].

### Appendix A.10. Scenario 10. SMARCA4-Deficient

Loss of *SMARCA4* defines an aggressive epigenetic subtype of LUAD. It is associated with poor prognosis and distinct immune features. Recent reviews summarize biology, co-alterations, and clinical behavior. They also highlight emerging synthetic-lethal strategies [78,79].

Genes and dysregulation: *SMARCA4* encodes the BRG1 ATPase, a component of the SWI/SNF complex. Truncating mutations and deletions cause protein loss. Co-mutations with *KRAS*, *STK11*, *KEAP1*, and TP53 are frequent and shape outcomes. Meta-analyses and focused reviews link *SMARCA4* loss to inferior survival and variable immunotherapy benefit [79,80].

Affected functions and pathways: Disruption of the SWI/SNF complex alters chromatin accessibility and lineage programs. Tumors adapt through metabolic rewiring and immune cold states [81,82].

Candidate therapeutic counteractions: Selective *SMARCA2* degraders show preclinical efficacy in *SMARCA4*-mutant lung models and are entering early trials. The reviews list multiple chemotypes and provide a rationale for clinical testing. Case-based and small clinical reports suggest activity for immunotherapy plus anti-angiogenic strategies in selected patients, but prospective validation is needed [81,82,83].

Counteraction vector: *SMARCA4*+, *SMARCA2*-, *EZH2*-, *CDK4*-, *CDK6*- [81].

Cross-cancer relevance: *SMARCA4* alterations appear in multiple solid tumors, including undifferentiated thoracic tumors and ovarian small-cell carcinoma. The same synthetic-lethal logic, as observed with *SMARCA2* and related chromatin targets, may apply across histologies [79].

## Appendix B

We reviewed the literature for all candidate treatments that passed our filtering and optimization. Most showed evidence of benefit, either directly or indirectly. However, some of them remain at the laboratory stage. Some are not specific to cancer therapy, while others have evidence in other cancers. These findings support the significance of our results and the relevance of the shortlisted compounds. In addition, detailed orthogonal validation results for each evaluated compound are provided in Supplementary File S6. It should be noted that CLUE does not necessarily include or adequately represent all compounds analyzed in this study.

1,2,5,8-tetrahydroxy anthraquinone: Quinalizarin (1,2,5,8-tetrahydroxyanthraquinone) shows antitumor activity (e.g., in A549 lung cancer by modulating AKT/MAPK/STAT3/p53 signaling, and in MCF-7 breast cancer by triggering ROS-mediated apoptosis via MAPK/STAT3/NF-κB) [84,85]. Formulation work using nanocellulose-alginate Pickering emulsions with diosgenin has been explored to deliver quinalizarin as a cytotoxic agent to lung/breast cancer cells [86]. Orthogonal CLUE-based transcriptomic reversal analysis did not provide direct support for this compound, because only indirect quinalizarin-related mapping was available.

2-chloro-5-nitrobenzanilide: 2-Chloro-5-nitrobenzanilide (GW9662) is an irreversible PPARγ antagonist (2-chloro-5-nitro-N-phenylbenzamide) used as a research tool to block PPARγ signaling. It covalently modifies the ligand-binding pocket, suppresses PPARγ-dependent transcription/adipogenesis, and modulates inflammatory and metabolic pathways relevant to cancer studies [87,88,89]. Orthogonal CLUE-based transcriptomic reversal analysis provided moderate support for this compound through the mapped perturbagen GW-9662.

3,5-Dihydroxy-4-methoxybenzyl alcohol: DHMBA (3,5-dihydroxy-4-methoxybenzyl alcohol) is an oyster-derived phenolic antioxidant that suppresses growth, migration, and invasion and promotes the death of metastatic prostate-cancer cells by targeting multiple signaling pathways [90]. Recent work also shows anticancer activity in bone-metastatic triple-negative breast cancer models [91]. Orthogonal CLUE-based transcriptomic reversal analysis did not provide direct support for this compound, because no direct CLUE perturbagen match was available in the current mapping.

Alisol B 23-acetate: Alisol B 23-acetate (AB23A) is a triterpenoid from Alismatis rhizoma that shows anticancer activity in NSCLC by suppressing PI3K–AKT–mTOR signaling and inducing apoptosis. It also modulates the tumor microenvironment by promoting M1 macrophage polarization via the regulation of CD11b/CD18. Overall, AB23A is considered preclinical/early translational and is not FDA-approved [92,93]. Orthogonal CLUE-based transcriptomic reversal analysis did not provide direct support for this compound, because no direct CLUE perturbagen match was available in the current mapping.

Allyl isothiocyanate: Allyl isothiocyanate (AITC), a pungent constituent of mustard, exhibits anti-cancer effects by inhibiting proliferation and migration. It also includes apoptosis in the lung and other tumor models [94]. Its chemistry, metabolism, and thiol conjugation shape bioavailability and activity [95]. Orthogonal CLUE-based transcriptomic reversal analysis did not provide direct support for this compound, because no direct CLUE perturbagen match was available in the current mapping.

Asparanin A: Asparanin A (a steroidal saponin from *Asparagus officinalis*) shows anticancer effects by arresting the cell cycle and triggering apoptosis (G_2_/M arrest in HepG2 hepatocellular carcinoma cells) [96]. In endometrial (Ishikawa) models, multi-omics profiling reveals G0/G1 arrest, reduced migration/invasion, and modulation of the Ras/ERK/MAPK and miRNA networks [97]. Orthogonal CLUE-based transcriptomic reversal analysis did not provide direct support for this compound, because no direct CLUE perturbagen match was available in the current mapping.

Bisdemethoxycurcumin: A turmeric curcuminoid with preclinical anti-inflammatory, anti-proliferative, metastasis suppression, and apoptosis induction effects modulating the PI3K/AKT and MAPK pathways [98,99]. Orthogonal CLUE-based transcriptomic reversal analysis provided strong support for this compound through the mapped perturbagen curcumin.

Cannabidiol: Purified CBD (as Epidiolex) is FDA-approved for seizures in Lennox–Gastaut syndrome, Dravet syndrome, and tuberous sclerosis complex. In addition, CBD enhances etoposide’s anticancer activity in NSCLC by p53-dependent inhibition of the PI3K–AKT–mTOR pathway, inducing autophagic cell death and suppressing oncogenes through a noncanonical mechanism. Moreover, studies in the literature have examined the therapeutic effects [100,101,102,103]. Orthogonal CLUE-based transcriptomic reversal analysis did not provide direct support for this compound, because no direct CLUE perturbagen match was retained in the current mapping.

Cladribine: Cladribine is a purine nucleoside analog that achieves high response rates in hairy cell leukemia and became a standard first-line option after short (5~7-day) courses [104]. Recent trials have also utilized cladribine as a backbone in combination with other therapies. For example, alternating cladribine with low-dose cytarabine and azacitidine plus venetoclax has shown strong efficacy in older acute myeloid leukemia (AML) patients [105]. Contemporary reviews reaffirm its central role in hairy cell leukemia (HCL) and outline its management [106]. Orthogonal CLUE-based transcriptomic reversal analysis provided strong support for cladribine in the current analysis.

Cordycepin: Cordycepin (3′-deoxyadenosine) shows broad anticancer activity, suppressing proliferation and migration in tumor models and reversing drug resistance, via kinase-pathway modulation (e.g., upregulating AMPK and downregulating AKT) and other mechanisms [107,108,109]. Recent reviews highlight the kinase-targeting potential and formulation/bioavailability advances of this compound and its antitumor effects [108,110]. Orthogonal CLUE-based transcriptomic reversal analysis provided moderate support for cordycepin in the current analysis.

Darinaparsin: Darinaparsin is a glutathione-conjugated organoarsenical with antitumor activity and mechanisms distinct from those of arsenic trioxide, involving mitochondrial/ROS stress and MAPK-linked signaling [111]. Clinical studies have tested IV and oral formulations in refractory solid tumors, and recent work shows that it can sensitize small-cell lung cancer to PARP inhibitors [112,113,114]. Orthogonal CLUE-based transcriptomic reversal analysis provided strong support for darinaparsin in the current analysis.

Dieckol: Dieckol, a phlorotannin from *Ecklonia cava*, has shown anticancer activity in pancreatic and colon cancer models [115,116]. Chemical derivatization (e.g., 6-O-acetyl dieckol) has been explored to enhance selective cytotoxicity against NSCLC cells [117]. Orthogonal CLUE-based transcriptomic reversal analysis did not provide direct support for this compound, because no direct CLUE perturbagen match was available in the current mapping.

DLBS 1425: DLBS 1425, a standardized *Phaleria macrocarpa* fruit extract, shows anti-proliferative and pro-apoptotic activity in breast cancer models, acting through PI3K/AKT downregulation and an eicosanoid-linked pathway [118]. Orthogonal CLUE-based transcriptomic reversal analysis did not provide direct support for this compound, because this extract-level entity was not directly represented in the current CLUE mapping.

Enzalutamide: Enzalutamide is a potent androgen receptor inhibitor with broad activity across various prostate cancer settings. Recent phase III data show that adding talazoparib to enzalutamide improves radiographic PFS in first-line mCRPC [119]. Contemporary reviews summarize its mechanism, resistance biology, and use in nonmetastatic CRPC [120,121]. Orthogonal CLUE-based transcriptomic reversal analysis provided strong support for enzalutamide in the current analysis.

Fenofibrate: An FDA-approved lipid-lowering fibrate, fenofibrate has shown oncology-relevant effects in preclinical models. It impairs the diapedesis of LUAD cells by disrupting a Cx43/EGF-dependent signaling axis. It also prevents cancer-associated muscle loss in lung cancer mice by redirecting hepatic metabolism [122,123]. Clinically, it remains indicated for dyslipidemia rather than cancer therapy. Orthogonal CLUE-based transcriptomic reversal analysis provided moderate support for fenofibrate in the current analysis.

GSK1210151A: GSK1210151A (I-BET151) is a pan-BET bromodomain inhibitor (BRD2/3/4) that blocks oncogenic transcription programs and shows antiproliferative activity across cancers, including NSCLC, where it suppresses growth partly via eIF4E downregulation [124,125]. Emerging reviews highlight I-BET151’s clinical challenges and optimization strategies (selectivity, combinations) within the BET-inhibitor class [126]. Orthogonal CLUE-based transcriptomic reversal analysis did not provide direct support for this compound, because no direct CLUE perturbagen match was retained in the current mapping.

HS 1200: HS-1200 is a synthetic chenodeoxycholic-acid derivative that triggers mitochondrial apoptosis and cell-cycle modulation in human hepatoma cells, partly via Egr-1-linked signaling [127]. In vivo, HS-1200 suppresses DEN-induced liver tumorigenesis and preserves liver function, supporting its potential as an antitumor agent [128]. Reviews further note HS-1200 as a bile acid analog with pro-apoptotic activity across various cancer models [129]. Orthogonal CLUE-based transcriptomic reversal analysis did not provide direct support for this compound, because no direct CLUE perturbagen match was available in the current mapping.

Ivermectin: In oncology, ivermectin shows preclinical activity in NSCLC, inducing apoptosis and nonprotective autophagy via *PAK1* downregulation. It can also enhance paclitaxel efficacy by overcoming *ABCB1*-mediated resistance. However, the evidence remains exploratory, and it is not FDA-approved for cancer treatment [130,131]. Orthogonal CLUE-based transcriptomic reversal analysis provided strong support for ivermectin in the current analysis.

Jinfukang: Jinfukang (a multi-herb TCM for NSCLC in China) is being tested to reduce chemotherapy-related adverse effects and improve outcomes in advanced disease [132]. Mechanistic studies suggest that it may inhibit metastasis by enhancing NK-cell-mediated clearance of circulating tumor cells [133]. Orthogonal CLUE-based transcriptomic reversal analysis did not provide direct support for this compound, because this multi-component formulation was not directly represented in the current CLUE mapping.

KR-62980: KR-62980 is a selective PPARγ agonist (SPPARM) with weak adipogenic effects that induces ROS-linked, proline-oxidase-mediated apoptosis in NSCLC cells. It remains a research tool, not FDA-approved [134,135]. This compound is formally known as 1-(methylimino-N-oxy)-6-(2-morpholinoethoxy)-3-phenyl-1H-indene-2-carboxylic acid ethyl ester. Orthogonal CLUE-based transcriptomic reversal analysis could not assign support for this compound because it was not represented in the current final CLUE summary table.

Metformin: Metformin, an antidiabetic drug, is under active repurposing in oncology; reviews link its anticancer effects to AMPK–mTOR and immune modulation [136,137]. In lung cancer, observational/meta-analytic signals of benefit contrast with a randomized trial in locally advanced NSCLC that found no added value with chemoradiotherapy [138,139,140]. Orthogonal CLUE-based transcriptomic reversal analysis provided strong support for metformin in the current analysis.

Motexafin gadolinium: Motexafin gadolinium (Xcytrin) is a redox-active gadolinium texaphyrin that localizes to tumors and enhances radiation effects, partly via ROS-mediated mechanisms [141,142]. In brain-metastases trials, adding it to whole-brain radiotherapy improved time to neurologic progression but did not yield regulatory approval, while imaging studies explored its dual optical/MR properties [143,144,145]. Orthogonal CLUE-based transcriptomic reversal analysis did not provide direct support for this compound, because no direct CLUE perturbagen match was retained in the current mapping.

Napabucasin: Napabucasin (BBI608) is an orally active naphthoquinone that inhibits cancer “stemness” largely via blockade of the STAT3 pathway with additional ROS-linked NQO1-dependent effects [146]. Preclinical and early clinical work across multiple tumors reports reduced proliferation, induction of apoptosis, and activity in select biomarker-defined groups. Meanwhile, class reviews outline ongoing challenges and combination strategies [147]. Orthogonal CLUE-based transcriptomic reversal analysis did not provide direct support for this compound, because no direct CLUE perturbagen match was retained in the current mapping.

NVP-AEW541: NVP-AEW541 is a selective small-molecule IGF-1R tyrosine-kinase inhibitor with strong in vitro and in vivo antitumor activity and marked IGF-1R and InsR cellular selectivity [148]. Recent work on nasopharyngeal carcinoma has shown that blocking IGF-1R with NVP-AEW541 disrupts IGF-1/AKT/S6 signaling and reduces bone metastasis features in models [149]. Orthogonal CLUE-based transcriptomic reversal analysis provided strong support for NVP-AEW541 in the current analysis.

Oleanolic Acid: Oleanolic acid, a natural triterpenoid, shows anticancer activity by blocking the purine salvage pathway in tumor metabolism and inducing PINK1-dependent mitophagy in A549 lung cancer cells (AKT-independent) [150,151]. Orthogonal CLUE-based transcriptomic reversal analysis provided moderate support for oleanolic acid in the current analysis.

Panduratin A: Panduratin A is a chalcone from *Boesenbergia rotunda* that shows anti-inflammatory activity via NF-κB/iNOS/COX-2 suppression. Additionally, it exhibits anticancer effects, including G_0_/G_1_ arrest and apoptosis, in NSCLC (A549) and hematologic models [152,153]. Sub-chronic oral dosing of a fingerroot extract enriched for panduratin A reported no serious toxicity up to 100 mg/kg/day in rats (formulated with cyclodextrin) [154]. Orthogonal CLUE-based transcriptomic reversal analysis did not provide direct support for this compound, because no direct CLUE perturbagen match was available in the current mapping.

Peracetylated N-azidoacetylmannosamine: Peracetylated N-azidoacetylmannosamine (Ac4ManNAz) is a bioorthogonal “azido sugar” used for metabolic glycan labeling to enrich/profile cancer-cell surface glycoproteins [155]. Variably acetylated ManNAz analogs can boost cell labeling efficiency compared with the classic tetra-acetate [156]. Recent work extends ManNAz-based labeling to glycoRNA on small extracellular vesicles [157]. Orthogonal CLUE-based transcriptomic reversal analysis did not provide direct support for this compound, because no direct CLUE perturbagen match was available in the current mapping.

Piroxicam: Piroxicam (a nonselective NSAID) shows repurposing potential in oncology. It modulates tumor-immune chemokines and NSAID-activated genes in colorectal cancer models. Oxicam reviews also link its antineoplastic effects to mitochondrial ROS elevation and apoptosis [158,159]. Broader surveys of anti-proliferative and anti-inflammatory agents also discuss piroxicam as part of repurposing strategies [160]. Orthogonal CLUE-based transcriptomic reversal analysis provided strong support for piroxicam in the current analysis.

Silymarin: Silymarin exhibits anti-cancer activity in lung models and reduces metastatic features. Studies implicate matrix metalloproteinase and motility pathways [161]. It also modulates MAPK signaling in NSCLC cells by curbing proliferation and invasion [162]. A review highlights apoptosis-focused mechanisms and notes nanodelivery strategies to improve its low bioavailability [163]. Orthogonal CLUE-based transcriptomic reversal analysis did not provide direct support for this compound because this mixture-level entity was not directly represented in the current CLUE mapping.

Solasodine: Solasodine shows anticancer activity by suppressing invasion and EMT signaling, e.g., in A549 lung cancer via miR-21/MMP downregulation, and in gastric cancer through AMPK/STAT3/NF-κB-mediated CLDN2 control [164,165]. Orthogonal CLUE-based transcriptomic reversal analysis did not provide direct support for this compound, because no direct CLUE perturbagen match was available in the current mapping.

SR 144528: SR 144528 (SR2) is a potent CB_2_ antagonist/inverse agonist widely used as a tool compound. In prostate cancer models, it helped attribute growth-inhibitory signaling to CB_2_ (blocking agonist effects in PC-3 cells) and reversed methanandamide-induced IL-6 secretion [166,167]. In lung cancer research, studies on cannabinoid effects (e.g., cannabidiol’s anti-invasive action) cite SR2 to investigate CB_2_ involvement in conjunction with *EGFR*-linked pathways [168]. Orthogonal CLUE-based transcriptomic reversal analysis provided moderate support for SR 144528 in the current analysis.

Staurosporine aglycone: Staurosporine aglycone (K252c; staurosporinone) is an indolocarbazole kinase inhibitor originally isolated (along with arcyriaflavin A) from a West African marine ascidian (*Eudistoma* sp.). It shows protein kinase C-linked cytotoxicity [169]. Subsequent work characterized rearranged K252c analogs from marine sponges by NMR [170]. Orthogonal CLUE-based transcriptomic reversal analysis provided strong support for this compound through the mapped perturbagen staurosporine.

Tanespimycin: Tanespimycin (17-AAG) is an HSP90 inhibitor that destabilizes oncogenic client proteins and shows anticancer activity across models and early trials [171,172]. In NSCLC, it can enhance the cytotoxicity of tamoxifen and erlotinib by downregulating thymidine phosphorylase via the inactivation of MKK1/2-ERK1/2. HSP90 blockade also disrupts RTK signaling, including ROR1. Orthogonal CLUE-based transcriptomic reversal analysis provided strong support for tanespimycin in the current analysis.

Telmisartan: Telmisartan is an AT_1_-receptor blocker with partial PPAR-γ activity. It also shows anticancer signals in lung models. Additionally, it inhibits A549 proliferation/migration via PI3K-AKT/mTOR downregulation and the induction of apoptosis [173]. Moreover, it sensitizes TRAIL by ROS-dependent DR5 upregulation through the inhibition of autophagy flux [174]. Orthogonal CLUE-based transcriptomic reversal analysis provided strong support for telmisartan in the current analysis.

Terpenes: A diverse class of plant and marine metabolites that exhibit anticancer signals across various models, including growth arrest, apoptosis, and metabolic rewiring. Recent reviews and studies have highlighted the therapeutic potential of terpene-rich extracts and identified specific terpenes (e.g., rosemary diterpenes and cannabis terpenes) that can inhibit tumor growth and enhance the efficacy of standard therapies. However, the translation is compound-specific [175,176]. Orthogonal CLUE-based transcriptomic reversal analysis provided strong support only at the mapped representative level through betulinic acid, because the broader terpene class was not directly profiled as a single CLUE entity.

Tetrahydropalmatine: Levo-tetrahydropalmatine (THP) shows anticancer potential. It targets the *hTERT* promoter G-quadruplex in NSCLC models. It suggests a route to telomerase suppression and induces apoptosis via mitochondrial ROS-driven metabolic switching in hepatocellular carcinoma [177]. A comprehensive review summarizes THP’s pharmacology and safety profile across indications [178]. Orthogonal CLUE-based transcriptomic reversal analysis provided moderate support for tetrahydropalmatine in the current analysis.

Tetrandrine: Tetrandrine, a bis-benzylisoquinoline alkaloid and calcium-channel blocker, shows anticancer activity in NSCLC by activating the STING/TBK1/IRF3 pathway, which enhances anti-PD-1 efficacy in vivo [179]. Nanoparticle delivery (platelet-membrane-coated tetrandrine) further boosts antitumor effects in NSCLC models [180]. Orthogonal CLUE-based transcriptomic reversal analysis provided weak support for tetrandrine in the current analysis.

Thapsigargin: Thapsigargin is a potent SERCA inhibitor that disrupts ER Ca^2+^ homeostasis, driving UPR-mediated stress and apoptosis in cancer cells [181,182]. To improve tumor selectivity, prodrug and delivery strategies, most notably the PSMA-activated prodrug mipsagargin, show preclinical and early clinical activity but remain investigational [183]. Orthogonal CLUE-based transcriptomic reversal analysis provided strong support for thapsigargin in the current analysis.

Triphenyl(phenylethynyl)phosphonium: Triphenyl(phenylethynyl)phosphonium (a TPP^+^-based mitochondria-targeting scaffold) is used to ferry anticancer payloads to the mitochondrial matrix. It can disrupt bioenergetics and amplify therapy response. Examples include mtDNA-targeting PIP-TPP conjugates that suppress A549 NSCLC growth in vivo and stearyl-TPP liposomes whose cytotoxicity tracks mitochondrial metabolism [184,185]. Orthogonal CLUE-based transcriptomic reversal analysis did not provide direct support for this compound, because no direct CLUE perturbagen match was available in the current mapping.

Vanillin: Vanillin (best known as a flavoring) shows anticancer signals. It suppresses migration/metastasis in vivo and inhibits CDK6 to curb proliferation in breast/lung models. Additionally, a vanillin-oxime derivative triggers JNK/ERK-CHOP-mediated apoptosis in NSCLC cells. A recent review summarizes pharmacology and safety across systems [186,187]. Orthogonal CLUE-based transcriptomic reversal analysis did not provide direct support for this compound, because no direct CLUE perturbagen match was available in the current mapping.

Zinc Acetate: Zinc acetate is an oral zinc salt that increases intestinal zinc and blocks copper absorption. It is FDA-approved for Wilson’s disease, not for cancer. In oncology research, zinc signaling influences tumor immunity and epithelial homeostasis. Reviews report context-dependent anticancer effects and epidemiologic links between zinc status and cancer risk [188,189,190]. Orthogonal CLUE-based transcriptomic reversal analysis did not provide usable direct support for this compound, because matching CLUE evidence was unavailable or not usable under the current mapping.
